# Promising approaches to support sustained colorectal cancer screening promotion strategies in primary care clinics

**DOI:** 10.1080/28322134.2025.2512477

**Published:** 2025-06

**Authors:** Dara Schlueter, Laura Arena, Cindy Soloe, Stephanie Melillo, Kate Ferriola-Bruckenstein, Esha Shah, Sonja Hoover, Florence K.L. Tangka, Sujha Subramanian

**Affiliations:** a Division of Cancer Prevention and Control, Centers for Disease Control and Prevention, Atlanta, GA, USA;; b RTI International, Durham, NC, USA;; c lmplenomics, Dover, DE, USA

**Keywords:** Colorectal cancer screening, primary care, evidence-based intervention (EBI), program evaluation, exploration preparation implementation sustainment (EPIS)

## Abstract

**Background:**

The Colorectal Cancer Control Program (CRCCP) funds recipients to partner with primary care clinics to adopt and sustain evidence-based interventions (EBIs) that increase CRC screening. This qualitative study explored how CRCCP recipients support their clinic partners to sustain EBI implementation.

**Materials and methods:**

Two waves of data collection – including 27 key informant interviews and 4 validation focus groups with CRCCP recipients – explored recipients’ preparation for partnering with potential clinics and supporting EBI implementation and sustainment. Thematic analysis identified support strategies used throughout the CRCCP lifespan.

**Result:**

To prepare for partnerships with clinics, recipients assessed organizational characteristics (leadership support, staffing, and data capacity) to determine readiness for implementing and sustaining EBIs. Recipients then: provided funding for implementation support, and ongoing training and technical assistance; established a clinic-level screening champion; and integrated EBIs into clinic workflows. Some recipients continue to partner with clinics after EBIs are sustained to monitor CRC screening rates and fund follow-up colonoscopies.

**Discussion:**

Study findings indicate that assessing organizational characteristics to determine readiness and providing funding and ongoing technical assistance are practical approaches to support CRC EBI sustainment. Results can inform program and partnership planning among CRCCP recipients and other cancer screening and chronic disease prevention programs.

## Introduction

Sustainable health systems-change approaches aim to increase the effects of public health initiatives by expanding the reach and quality of healthcare – beyond the individual patient to investment in systematic changes to health policies, practices, and structures [1–3]. These models may also address health disparities by implementing evidence-based strategies among medically underserved populations. Recent focus on enhancing these practices emphasizes the importance of multi-sectoral collaboration – including partnerships between public health and primary care – to drive progress towards public health goals [4,5]. This cross-sector integration increases the capacity of each entity while maximizing their collective impact on population health [6].

The Centers for Disease Control and Prevention (CDC)’s Colorectal Cancer Control Program (CRCCP) uses a health systems-change model that promotes public health-primary care partnerships to address disparities in colorectal cancer (CRC) screening. CRC screening is effective for reducing CRC mortality [7]. However, screening disparities exist among certain groups, such as people who are uninsured [8]. In 2020, the CRCCP funded 35 recipients – including state health departments, universities, tribal organizations, and other organizations – to partner with primary care clinics to implement evidence-based interventions (EBIs) recommended by The Community Preventive Service Task Force described in the Guide to Community Preventive Services (the Community Guide) [9]. The aim was to increase CRC screening in clinics. CRCCP recipients support clinic partners (who are mostly federally qualified health centers [FQHCs]) to adopt and enhance EBIs that are sustainable. Sustainability was defined as fully integrated into health system or clinics’ ongoing operations, coupled with high-quality implementation and the necessary supporting infrastructure to maintain implementation [10]. Implementing high-quality EBIs that are sustainable supports effective, long-term systems change that can continue if time-limited funding becomes unavailable.

The CRCCP has had success in supporting implementation and sustainment of EBIs. During the previous funding cycle (2015–2020), CDC analyzed the relationships between EBI implementation and sustainment and clinic enrollment time. The analysis showed that as the duration of clinics’ enrollment in the CRCCP increased, both EBI implementation and sustainment also increased [10]. Previous research identified factors that support EBI sustainment, including clinic leadership support and integration of EBIs into clinic workflows and procedures [11]. However, there is limited knowledge about how these and other programmatic factors are operationalized in real-world settings.

This study explored how CRCCP recipients support their partner health systems and clinics to facilitate EBI sustainment. For the purpose of this study, we define sustainment as implementation of EBIs in partner health system and clinic settings that could be continued and maintained following the initial funding period. This practice-based evidence can be used by CRC screening promotion programs and other chronic disease prevention initiatives to inform cross-sector partnerships – to support EBI sustainment and for continuous program improvement.

## Materials and methods

**Figure 1.**
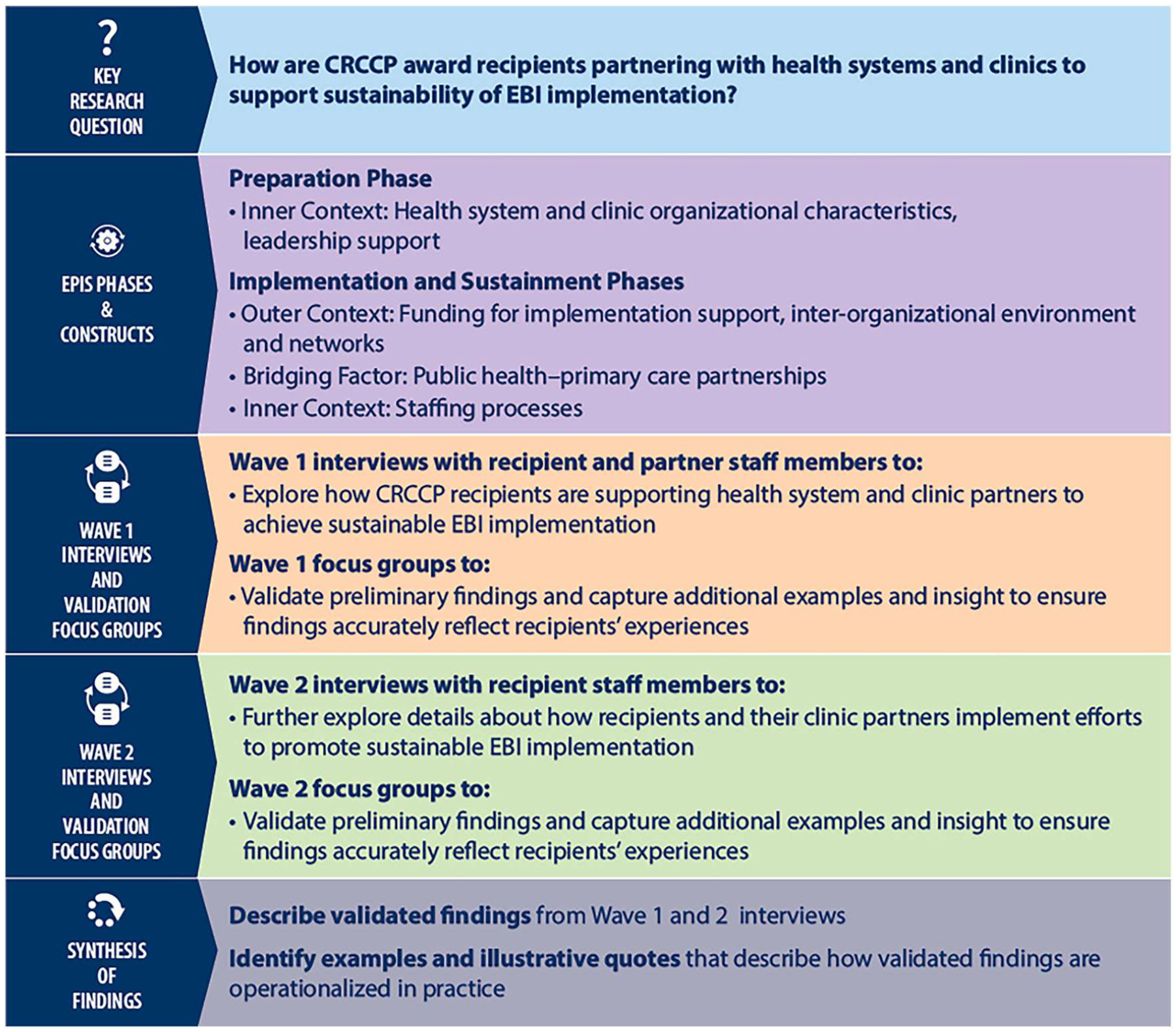
Overview of methods.

We conducted a qualitative multi-case study, which included two waves of data collection, to answer our primary research question: ‘How are CRCCP award recipients partnering with health systems and clinics to support sustainment of EBI implementation?’ In Wave 1, we explored how CRCCP recipients are supporting their health system and clinic partners to achieve sustainment of EBIs that promote CRC screening. Wave 2 built on main findings from Wave 1 and focused on eliciting details about how recipients and their clinic partners implement efforts to promote sustainability. Figure 1 presents an overview of our study methods.

### EPIS framework

We used constructs from the Exploration, Preparation, Implementation, Sustainment (EPIS) Framework [12], an implementation science framework that has been widely used to evaluate EBI implementation across many settings [13]. EPIS was used to guide development of the evaluation questions and data collection tools. We selected EPIS constructs that aligned with CRCCP program components and that could be feasibly assessed through qualitative inquiry. Table 1 presents relevant EPIS constructs, aligned with interview topics, aimed at understanding how each construct influenced recipients’ strategies for supporting sustainment of EBI implementation.

### Recipient selection

We used purposive sampling to select nine CRCCP award recipients for participation in this study. We selected recipients to reflect the diversity across CRCCP and considered several program characteristics, including priority populations served, geographic region, participation in previous CRCCP funding, and type of recipient organization (Table 2). We also considered recipients’ implementation practices, including type of support provided to health system and clinic partners (i.e. direct support or training and technical assistance [TTA] through partner organization). Finally, we considered the proportion of each recipients’ clinic partners that reported achieving at least one sustainable EBI for at least 2 consecutive years, which ranged from 6% to 100% with an average of 67%. Recipients identified two participants from their organization or from a partner organization to participate in the study: one involved in supporting health systems and clinic partners with CRC EBI implementation, and one involved in evaluation of EBI implementation.

### Data collection

During March – June 2023, we conducted 26 virtual interviews with CRCCP recipient, implementation, and evaluation staff representing the nine selected recipient organizations (Wave 1). In December 2023, we conducted nine follow-up interviews with CRCCP recipient representatives, all but one of whom also participated in Wave 1 (Wave 2; Table 3).

We developed unique interview guides for each respondent type based on EPIS phases relevant for this study (i.e. Preparation, Implementation, Sustainment). After synthesizing Wave 1 interview findings, the study team identified themes and content areas for deeper exploration in the Wave 2 CRCCP recipient interview guide. Wave 2 focused on operationalizing findings from Wave 1. For example, in Wave 1 we broadly explored how recipients use readiness assessments to support sustainability planning with health system/clinic partners and learned that some recipients conduct ongoing assessments throughout the project period. In Wave 2, we focused on understanding the frequency, topics, and processes for conducting initial and ongoing assessments throughout the project period.

For each Wave, 60-minute interviews were conducted by an interviewer and notetaker. Interviewers were qualitative researchers, experienced in conducting interviews across multiple studies. Interviewers and notetakers were also involved in developing and refining the data collection instruments. All participants gave verbal consent to participate in interviews and to be audio-recorded. Recordings were transcribed for analysis. Institutional Review Board approval was not required for this data collection because it did not constitute human subjects’ research.

After each Wave of interviews, the study team conducted two virtual focus groups with the CRCCP recipient representatives who had participated in an interview. Participants were offered two timeslots and given the option to choose the one most convenient for them. Wave 1 focus groups were conducted in July and August 2023, and the Wave 2 focus groups were conducted in February and March 2024. The study team developed focus group moderator guides to (1) present preliminary themes to recipients, (2) gather feedback on themes reflecting recipients’ common strategies for supporting health system and clinic partners to sustain EBI implementation, and (3) probe on real-world examples, barriers, and facilitators to implementing these strategies. At the end of each focus group, participants were asked to rank the top three programmatic factors that were most important for EBI sustainment from a list of ten factors that had emerged from the current study and previous research [11] (see Supplementary Material C).

### Analysis

The study team conducted rapid qualitative analysis to identify preliminary themes. A lead notetaker documented details during each interview in an Excel spreadsheet that mapped participant responses to interview guide topics. A team of three analysts: (1) abstracted interview data from the Excel spreadsheet into a document that summarized preliminary themes, (2) referred to interview transcripts as necessary for clarity or examples of each preliminary theme, and (3) met to refine the list of preliminary themes. Findings that reflected input from three or more recipients were identified as ‘themes’. Promising findings that reflected input from two recipients were noted as ‘emerging themes’. Themes and emerging themes were validated with recipient representatives during focus groups. The team used focus group findings to validate, build on, and operationalize our findings with specific examples from interviews. Two analysts conducted a primary and secondary review of detailed notes from each focus group to document participants’ examples of agreement and disagreement with each strategy, and to identify common facilitators and challenges related to each. A third analyst conducted a final review and adjudication of validated themes which are presented in the results.

## Results

**Figure 2.**
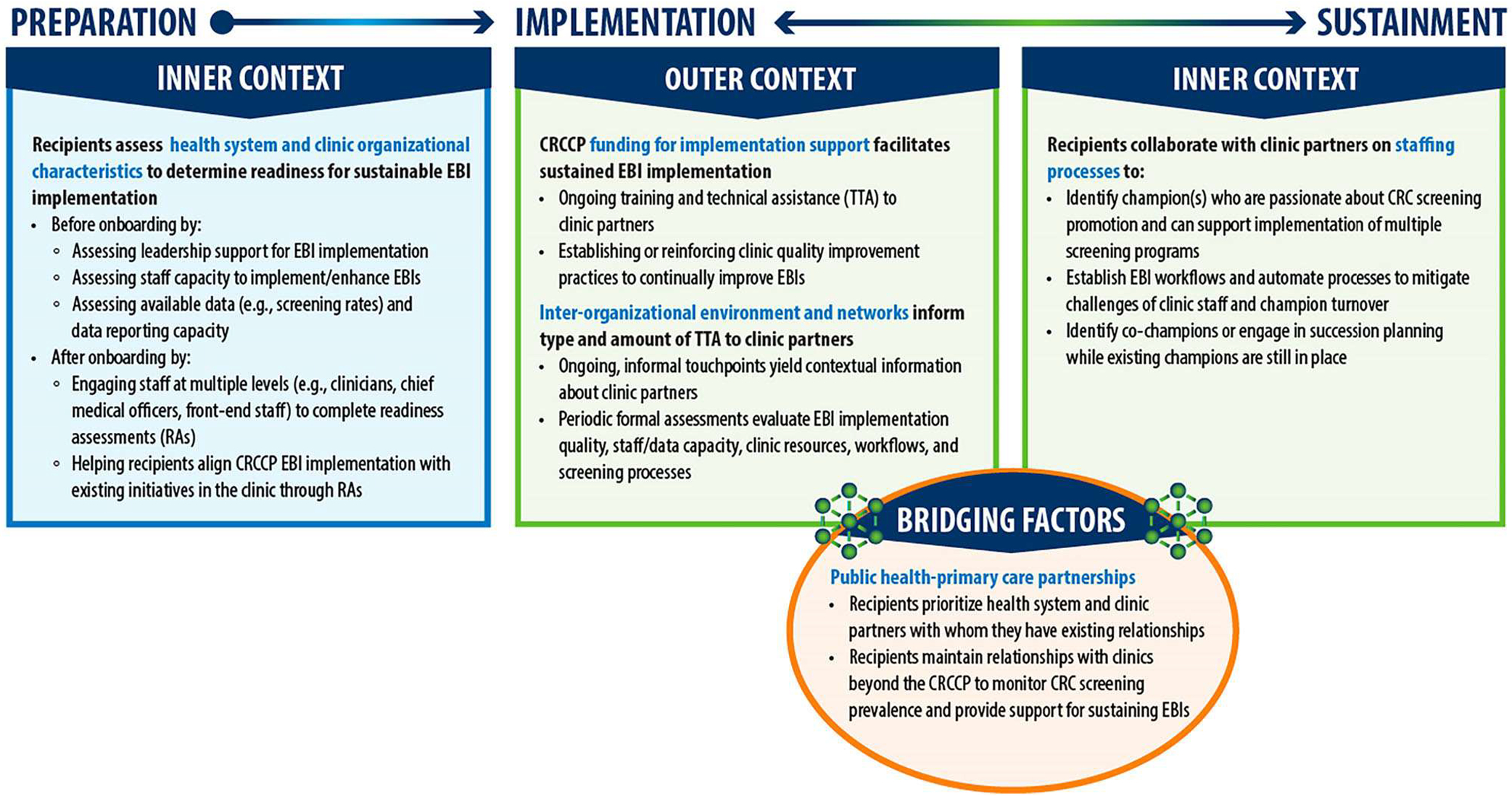
Key findings by EPIS phase and construct. *EPIS = Exploration, Preparation, Implementation, Sustainment (EPIS) Framework; EBIs = evidence-based interventions; RA = readiness assessment; CRCCP Colorectal Cancer Control Program; TTA = training and technical assistance. *Blue text reflects EPIS constructs.

We identified five themes from data analysis that reveal how recipients collaborate with health system/clinic partners to support sustainable EBI implementation during the preparation, implementation, and sustainment phases. Figure 2 presents an overview of key qualitative findings by EPIS construct; the five identified themes appear in blue text. In addition, respondents ranked (1) institutionalizing EBIs into standard operating procedures, (2) implementing CRC EBIs with other chronic disease initiatives, and (3) clinic leadership support for EBI implementation as the top three factors that support EBI sustainment. In the following sections, we further describe each theme and provide illustrative respondent quotes. Additional supporting data are provided in Supplementary files A, B, and C.

### Health system and clinic organizational characteristics

During Wave 1 interviews and focus groups, respondents described how readiness assessments can set the stage for sustained EBI implementation. Some recipients described engaging staff at multiple levels (e.g. clinicians, chief medical officers, front-end staff) in the assessment to encourage a team-based approach to EBI implementation from the beginning of the partnership. Respondents explained that this helps ensure that staff at multiple levels: (1) are informed and invested in the CRCCP and (2) can maintain EBI implementation should there be clinic experience staff turnover. Readiness assessments also help recipients align CRCCP EBI implementation with existing clinic initiatives and facilitate adoption of a sustainable workflow that aligns with how clinic care teams function. Respondents described their approaches to assessing readiness before and after onboarding clinic partners.

Prior to partnering with clinics, recipients reported conducting an initial vetting of potential health system and clinic partners to ensure they have leadership support, as well as sufficient staff and data capacity, to implement and sustain CRC EBIs. During Wave 1 interviews, respondents emphasized the importance of engaging leadership to facilitate EBI sustainment. Additionally, during Wave 2 focus groups, respondents were asked to rate various factors that support EBI sustainment, from most important to least important. Ranked as the top two most important factors were institutionalizing EBIs into standard operating procedures, and having leadership support. Recipients acknowledged the critical role of leadership support in facilitating implementation and sustainment. Recipients also described having informal meetings with health system/clinic leaders to determine whether they could identify a point of contact to advocate for the work. One respondent stated:

- *I think ultimately one of the main goals is buy-in from leadership on the importance of utilizing and institutionalizing those EBIs… The leadership plays a crucial role in the funding and staffing needed to properly implement those EBIs.* – **Wave 1 Interview, Recipient 5, Health Department**

Another respondent noted that they would not pursue a partnership with a clinic that is unable to identify a staff member to champion the work.

- *Before we decide to take on a partner site for EBI implementation, those are the conversations we have specifically with leadership… if they can identify someone, a point of contact and someone who can drive this forward. If those answers are no, we would not enter into a partnership with that site.* – **Wave 2 Interview, Recipient 6, University**

Although recipients described different methods for vetting potential partners, they shared a common goal of assessing clinic staffing and data capacity, both to meet reporting requirements and to gauge interest in CRCCP participation. Two recipients described having multiple conversations with potential clinic partners before obtaining letters of support for the CDC funding application. One recipient described using a competitive request for applications process, which required potential clinic partners to (1) report their CRC screening rates and (2) answer questions about their patient population, data reporting processes, and staff members to be responsible for implementing and strengthening EBIs. Another recipient described reviewing clinic-level screening rates and uniform data system (UDS) data^1^ before sending an invitation letter to potential clinic partners. One recipient described assessing the clinic’s ability to ensure that patients with a positive stool-based test would receive a follow-up colonoscopy.

- *We reach out to them to invite them to participate. We look at their screening rates, we look at their UDS reporting for the whole health system to see what their screening rates are, and then we send a letter describing what would be involved in this kind of program – and then ask them if they would like to participate.* – **Wave 2 FG, Recipient 9, University**
- *We put out a bid. So our applicants, clinics, have to apply for the funding… They have to report on their screening rates… We ask them about their ability to report out on ordered and completed FIT [fecal immunochemical test] screening diagnostics… They have to name the staff that they’re going to put on this work… That weeds out clinics that we just don’t think are able to fully participate.* – **Wave 2 FG, Recipient 4, Health Department**

Once clinic partners are onboarded to the CRCCP, before they transition to the Implementation Phase, all recipients reported conducting in-depth readiness assessments. Readiness assessments examined the following topics: presence of a clinic champion; clinic resources and capacity for EBI implementation (e.g. clinic staff support, leadership support, space and materials); electronic health record (EHR)’s capacity to generate CRC screening rates and support EBI implementation; workflow and screening processes; clinic’s method for calculating CRC screening rate; EBIs being implemented or enhanced; and clinic staff training needs.

Recipients acknowledged that the formal readiness assessment process can be challenging for busy clinic staff. To minimize that burden, respondents recommended that recipients shorten their readiness assessment tool, extract screening rate data on behalf of clinics if possible, and/or complete the assessment via verbal conversation with clinic staff.

- *Some clinics prefer to talk to fill out some of the more in-depth sections of the readiness assessment tool. We found that to be useful and not as burdensome on them. Some people would rather have a conversation than type things out.* – **Wave 1 FG, Recipient 2, Other**

### Funding for implementation support

Interview and focus group respondents stated that funding for continuous quality improvement (QI) and TA was important for implementation support and sustainment. During Wave 2 interviews, three recipients emphasized elements that were important for sustainment of EBIs: (1) continued allotment of clinic funds to support EBI implementation and (2) ongoing contact with clinics. When asked to describe the reality of sustainment as envisioned in the CRCCP model, recipients reported that ongoing touchpoints are necessary to promote accountability.

- *Clinics wanted to partner with us again because we were holding them accountable. They said since we left, [their] rates have dropped and when they work with us, they were more accountable to meeting quality measures.* – **Wave 2 Interview, Recipient 9, University**

Recipients use funding to provide implementation support to clinics and build QI practices to support sustainment of EBI implementation. Two recipients in the Wave 1 focus groups reported that establishing and reinforcing QI practices in the first year supports informed selection of EBIs, EBI implementation, and sustainment of EBIs.

1. *We said, no, we’re not implementing EBIs in the first year. We’re doing QI for the first year [and] they’ll pick their EBIs by the last couple of months of the first year because they need to take the time to look at what they’re doing [so]that quality improvement can be sustainable.* – **Wave 1 FG, Recipient 7, University**

### Inter-organizational environment and networks

During Wave 2 focus groups, recipients reported providing ongoing TTA to help clinic partners sustain EBI implementation. When asked to rank factors that support EBI sustainment, recipients ranked ongoing TTA as the third most important factor for supporting EBI sustainment. To help inform future TTA, some recipients discussed conducting ongoing, informal touchpoints with clinic partners to gather contextual information about factors affecting CRCCP implementation. In addition, recipients reported using various benchmarks/milestones (e.g. clinic-level screening rates) to (1) inform the TTA they provide to clinic partners, and (2) decide to taper or increase the amount of TTA they provide.

- *So in the informal meetings it’s interesting because it’s not necessarily like the topic is CRCCP, you know, it’s kind of just like, how are things going? And so you’ll get a variety of answers that can be staff turnover, adopting this new technology that we wouldn’t have found out about until much later on. And by understanding what’s going on in the clinics. It just provides a lot of information and a lot of things that you can go off and help with.* – **Wave 2 FG, Recipient 1, Other**

Throughout interviews and focus groups, respondents noted the importance of having ongoing touchpoints with clinic partners as a means to support sustained EBI implementation. For example, they discussed the importance of revisiting readiness assessment data.

- *Assessment tools are a starting point, but you have to continually check in. At the end of the day, when you are assessing readiness, you have to continue going back. There is incredible turnover. That is the nature of FQHCs. We always assess if there have been changes to capacity or readiness.* – **Wave 1 FG, Recipient 7, University**

Recipients also reported conducting formal periodic assessments (e.g. quarterly) with clinic partners to examine EBI implementation quality; staff and data capacity; clinic resources, workflows, and screening processes; and/or other TTA needs. Recipients stated that expectations around formal assessments were clearly communicated early in the partnerships, so clinics knew what to expect. In addition, recipients conducted recurring meetings with clinic partners to follow up on specific areas of improvement that emerged as priorities during periodic assessments.

- *Our formal assessments ask about workflows, health information technology capacity. And once that information is collected, our staff go in and work with the clinics to say, this is what you articulated to us in your assessment. Now let’s look at it as a process map. Does this look right to you? Where can we begin improvement?* – **Wave 2 FG, Recipient 9. University**

### Staffing processes

During Wave 2 interviews and focus groups, respondents emphasized the role champions play in ensuring that CRC screening remains a focus among competing clinic priorities. Respondents provided examples of how to select an effective champion and strategies for maintaining EBI implementation when a champion is lost due to staff turnover. Some respondents reported that supporting clinic partners in selecting a clinic champion who is familiar with the CRC patient population – and passionate about CRC screening promotion – facilitates EBI implementation and sustainment. Respondents from these sites noted that an effective champion can support implementation of multiple screening programs and/or disease types, and that success in one area can lead to improvements in another.

- *Every clinic champion that I know keeps the CRC in the forefront of their clinic space … they’re like the ringleader. They make sure that everybody is still thinking about it [and] if there’s a new staff person, that they’re aware that CRC is a focus of that clinic, and that everybody is trained on the processes and workflows for their CRC screening processes.* – **Wave 2 interview, Recipient 8. Health Department**

Respondents from two sites reflected an emerging theme of succession planning while existing champions are still in place. For example, a respondent from one site mentioned identifying ‘co-champions’ so that if one champion leaves, the other champion could continue prioritizing EBI implementation until another co-champion is identified.

- *Empowering whoever the current champion is to think long term about who can they bring up alongside them so that if they do transition, or when there is turnover, there’s an easy succession.* – **Wave 2 FG, Recipient 3, University**

### Public health-primary care partnerships

Recipients described having long-term relationships with their health system and clinic partners that were established through previous CRCCP funding periods or other health promotion/chronic disease prevention initiatives. Recipients reported that these existing relationships help ensure strong partners that facilitate CRCCP implementation and sustainment.

- *We go to folks we’ve worked with before or through our work with the breast or cervical screening program. We know those clinics. We know their capacity to some degree. So I think that’s how we utilize that information to prioritize who we reach out to [for CRCCP].* – **Wave 2 FG, Recipient 9, University**

Recipients also reported a variety of reasons for maintaining clinic partnerships beyond EBI sustainment. These reasons include: monitoring CRC screening prevalence; providing support for sustaining EBIs through clinic changes (e.g. staff turnover, EHR changes); providing ongoing funding for follow-up colonoscopies; supporting workforce development; and adding or enhancing additional EBIs. Respondents noted that continuing periodic check-ins with clinic partners is necessary to help clinics maintain their target CRC screening rates over time.

- *I feel like it’s a living organism, you know what I mean? It’s never really like, check, that EBI is done. So once they’ve reached sustainability where we feel like we’ve gone as far as we can with working with them on this particular EBI, [we need] to continue oversight, and to continue supporting changes that may impact that EBI. That’s how we work*. – **Wave 2 Interview, Recipient 5, Health Department**

## Discussion

This qualitative study identified promising approaches for facilitating sustainment of EBIs within health systems and primary care clinics. These nine CRCCP recipients provide real-world examples of how relevant EPIS framework constructs may be operationalized in CRC screening programs, and how these theoretical factors function throughout the lifespan of public health-primary care partnerships. Findings provide practice-based evidence to inform specific strategies to foster partnerships between CRC screening programs (public health) and health systems and clinics (primary care) aimed at implementing and sustaining CRC screening promotion initiatives. Respondents’ rankings of the most important program factors provide additional context and reinforcement of the value of the strategies described to sustain EBIs.

It is important for organizations to establish their readiness by demonstrating commitment and capacity for change before adopting a new systems-level change approach [14]. Recipients’ initial vetting of potential partners helped determine whether clinics had the basic capacity to implement the CRCCP (e.g. leadership support, high-quality CRC screening data). Full readiness assessments provide insight into capacity around programmatic factors that support readiness [15] such as: clinics’ staffing capacity; EHR systems and capacity to collect high-quality CRC screening data; EBI implementation practices; and existing clinic workflows. These steps in preparation support efficient selection of appropriate clinics; however, clinics that have high interest but lack capacity (e.g. unable to report required CRC screening data or use EHR workflow processes) may be overlooked for partnership. Such clinics would therefore miss the opportunity to implement and sustain EBIs to increase CRC screening among their patient populations. Future research may explore the capacity building needed to prepare these clinics to participate in the CRCCP, and optimal approaches to providing that support.

Throughout implementation, CRCCP recipients focused largely on facilitating continuous QI in clinics. Periodic touchpoints – to conduct informal assessments of clinics’ needs and provide appropriate TA – established and reinforced clinics’ QI processes while also strengthening two-way accountability between partners. Recipients’ flexibility in how ongoing support was provided – such as shifting the TA frequency and content to accommodate clinic priorities – reinforced the importance of meeting clinics where they are. Previous studies highlight the critical nature of meeting partners where they are to strengthen collaboration and facilitate long-term implementation [11,16–20]. This study provides multiple real-world examples of this key tenet of public health service in action.

Partnerships between recipients and health systems/clinics can serve as bridging factors between the inner and outer contexts in which EBIs are implemented. CRCCP recipients’ partnerships with health systems and clinics are intended to be timebound; partnerships with clinics typically end once clinics meet a CRC screening rate benchmark (e.g. greater than 60%) and/or sustainable EBIs are in place. Many recipients have longstanding partnerships with health systems and clinics that span multiple funding cycles. However, this study revealed that some recipients continue to partner with clinics even after EBI sustainment to help monitor CRC screening rate changes, support continued EBI implementation during periods of clinic change, and provide resources for follow-up colonoscopy. This may reveal the need for longer term support to truly enable clinics to sustain EBIs beyond the CRCCP funding cycle. Future studies can explore opportunities to provide lengthier, nonfinancial support (e.g. training, TA) needed to ensure that clinics are well prepared to sustain EBIs and other CRCCP activities over time.

The value of having an established clinic-level champion for CRC screening uptake is well documented [17, 20]. This study contributes to our understanding of how health systems and clinics can approach identifying appropriate champions. Ideally, champions are not only passionate staff who have the interest and capacity to advocate for CRC screening, they are also familiar with the clinic’s patient population so they can influence EBI implementation and sustainment in their clinic. To maximize the champion’s value over time, this study highlights the importance of succession planning to maintain the impact of the champion in the event of staff turnover. Champions are not only important for initiating the program but are also essential for ongoing implementation and sustainment as new priorities and challenges emerge. By embedding the champion role in the clinic structure – such as incorporating champion responsibilities into specific staff descriptions and establishing co-champions that work collaboratively to facilitate a seamless transition when there are staffing changes – clinics may sustain the value of champions beyond the service of any one clinic staff person.

There are at least three study limitations. First, this is a qualitative study; although study findings are not generalizable, they could be applicable in similar settings and programs. Second, findings represent a small sample of CRCCP recipients and therefore reflect the experiences and opinions of a relatively small number of study participants. This limitation was mitigated by our purposive approach to selecting recipients that were representative of different geographic regions, populations served, and proportion of clinic partners that had achieved EBI sustainment. Third, although we completed two waves of data collection to conduct a deep exploration of key themes, we did not prospectively follow the recipients and their partner clinics over time to capture processes related to preparation, implementation, and sustainment. Fourth, this study focused on current CRCCP recipients only; therefore, it was outside of the study scope to measure EBI implementation following the current funding period.

## Conclusion

We used a theoretical framework to explore potential strategies for supporting primary care clinics in implementing sustainable CRC EBIs. Results provide real-world examples of how CRC screening programs can strengthen public health-primary care partnerships to facilitate CRC screening promotion activities – throughout the preparation, implementation, and sustainment phases of a systems-level change initiative. These examples can be used by CRCCP recipients, other CRC screening programs, and chronic disease prevention initiatives to inform program and partnership planning efforts. Future research can explore strategies for building capacity among health systems and clinics that lack the capacity to implement the CRCCP.

## Supplementary Material

Schleuter_Supplement A

Schleuter_Supplement B

Schleuter_Supplement C

Supplemental data for this article can be accessed online at https://doi.org/10.1080/28322134.2025.2512477

## Figures and Tables

**Table 1.** Relevant EPIS constructs, definitions, and related interview topics.

Theory construct and definition	Interview topics Wave 1	Interview topics Wave 2
**Preparation Phase**		
**Inner Context: Organizational Characteristics** *Structures or processes that take place and/or exist in organizations that may influence the process of implementation (e.g. culture, climate, readiness for change, structure, leadership, receptive context, absorptive capacity, social network support)*.	How do recipients use readiness assessments (or other planning assessments) to support sustainability planning with health system/clinic partners?	To what extent do recipients conduct ongoing assessments with health system/clinic partners? Which domains are most important to assess readiness for sustainable EBI implementation? What do health system/clinic partners – without the capacity to participate in CRCCP – need to become ready?
**Inner Context: Leadership** *Characteristics and behaviors of individuals involved in oversight and/or decision-making related to EBI implementation within an organization*.	How important are the following factors for supporting sustainment of EBIs: Providing ongoing support for optimal EHR use Engaging clinic leadership Adopting a team-based approach and cross-training clinic staff Institutionalizing EBI implementation into existing workflows Integrating implementation of EBIs to promote CRC screening with other screening and chronic disease management activities	
**Implementation & Sustainment Phases**		
**Outer Context: Funding for Implementation Support** *Funding includes fiscal support provided by the system in which implementation occurs. Fiscal support can target multiple levels (e.g. staff training, fidelity monitoring/continuous quality improvement [QI])*.	What role does funding play in helping health system/clinic partners sustain EBI implementation? What training/technical assistance do recipients provide to help health system/clinic partners sustain EBI implementation?	What training/technical assistance do recipients conduct with health system/clinic partners around QI to support EBI implementation? How does QI support sustainment of EBIs?
**Outer Context: Inter-Organizational Environmental Networks** *Relationships of professional organizations through which knowledge of the EBI is shared and/or goals related to the EBI implementation are developed/established*.	To what extent do recipients establish expectations around sustainment of EBI implementation at the time of enrollment? How do they do that?	How do recipients support clinic partners in sustaining EBI implementation and CRC screening rates?
**Bridging Factors: Partnerships** *Bridging factors influence the implementation process as the inner context of organizations is influenced by the outer system in which the organization operates, and those influences are reciprocal. Bridging factors may include processes such as interagency collaboration and community-academic partnerships*.	Why and how do recipients continue or end relationships with health system/clinic partners?	What oversight and support do recipients provide to health system/clinic partners once they have sustained CRC EBIs? To what extent is it realistic for recipients to step away from clinic partners and for clinics to sustain EBIs and maintain or continue to improve CRC screening rates?
**Inner Context: Staffing Processes** *The processes or procedures in place at an organization related to the hiring, review, and retention of staff involved in the active delivery of the EBI and/or its implementation (e.g. professional training and qualification related to EBI delivery, staff turnover)*.	How important are the following factors for supporting EBI sustainment: Providing ongoing support for optimal EHR use Engaging clinic leadership Adopting a team-based approach and cross-training clinic staff Institutionalizing EBI implementation into existing workflows Integrating implementation of EBIs to promote CRC screening with other screening and chronic disease management activities	How do champions help sustain EBI implementation?

EPIS = Exploration, Preparation, Implementation, Sustainment (EPIS) Framework; CRCCP = Colorectal Cancer Control Program; CRC = colorectal cancer; EBI = evidence-based intervention; EHR = electronic health record; QI = quality improvement.

**Table 2.** Summary of participating recipient characteristics.

	Priority Population(s) Served			U.S. Region				Previous CRCCP Funding Cycle		Type of Recipient Organization		
Recipients	Racial/ ethnic minority groups as priority population	Rural	Urban	NE	MW	S	W	Yes*	No	Health Dept	University	Other^+^
Recipient 1	•	•					•		•			•
Recipient 2	•		•				•	•				•
Recipient 3	•	•	•			•			•		•	
Recipient 4	•	•	•	•				•		•		
Recipient 5		•	•	•				•		•		
Recipient 6	•	•	•		•			•			•	
Recipient 7	•	•	•			•		•			•	
Recipient 8	•	•	•				•	•		•		
Recipient 9		•				•		•			•	
Totals	**7**	**8**	**7**	**2**	**1**	**3**	**3**	**7**	**2**	**3**	**4**	**2**

CRCCP = Colorectal Cancer Control Program; NE = Northeast; MW = Midwest; S = South; W = West.

* Note: Participated in CRCCP from 2015 to 2019 (DP15-1502).

+ Note: Other organization types included a health system and state primary care association.

**Table 3.** Number and type of respondents.

	Wave 1			Wave 2	
Recipient	Program Director/Manager *(Recipient Staff)*	TA Provider *(Recipient/Partner Staff)*	Evaluator *(Recipient/Partner Staff)*	Program Director/Manager *(Recipient Staff)*	Total Unique Interviewees*
Recipient 1	1	1	1	1	3
Recipient 2	1	1	1	1	3
Recipient 3	1	1	1	1	3
Recipient 4	1	1	1	1	3
Recipient 5	1	1	1	1	3
Recipient 6	1	1	1	1	4
Recipient 7	1	1	1	1	3
Recipient 8	1	0	1	1	2
Recipient 9	1	1	1	1	3
**TOTAL**	**9**	**8**	**9**	**9**	**27**

TA = technical assistance.

* Note: The study team conducted interviews with a total of 27 unique respondents across Waves 1 and 2, including 26 unique respondents in Wave 1, and 1 additional unique Program Director/Manager respondent who had recently assumed the position in Wave 2.

## Data Availability

The qualitative data generated or analyzed during the current study are not publicly available because they were generated in interviews and focus groups conducted by the research team, with the expectation that participant identity would be kept confidential.

## Footnotes

1 Federally Qualified Health Centers that receive funding from the Health Resources & Services Administration are required to report a core set of information, including data on patient characteristics, services provided, clinical processes and health outcomes, patients’ use of services, staffing, costs, and revenues as part of a standardized reporting system known as the Uniform Data System (UDS). Source: https://data.hrsa.gov/tools/data-reporting/program-data
