# Supplementary material for: Promising approaches to support sustained colorectal cancer screening promotion strategies in primary care clinics
Source: Prev Oncol Epidemiol. Author manuscript; Available in PMC 2025 Sep 9. (PMC12416315; doi:10.1080/28322134.2025.2512477)
Supplement: Schleuter_Supplement A [file NIHMS2099663-supplement-Schleuter_Supplement_A.docx]

**Supplementary Material A: Key Findings by EPIS Phase and Construct**

| **EPIS Phase/Construct** | **EPIS Definition** | **Findings (from Interviews, Focus Groups)** |
| --- | --- | --- |
| **Preparation** | | |
| Inner Context: Health System and Clinic Organizational Characteristics, Including Leadership Support | - Organizational Characteristics: structures or processes that take place and/or exist in organizations that may influence the process of implementation (e.g. culture, climate, readiness for change, structure, leadership, receptive context, absorptive capacity, social network support) - Leadership: characteristics and behaviors of individuals involved in oversight and/or decision-making related to EBI implementation within an organization | - Recipients assess health system and clinic organizational characteristics to determine readiness for sustainable EBI implementation. - Before onboarding by:   - Assessing leadership support for EBI implementation (Wave 2 FG)   - Assessing staff capacity to implement/enhance EBIs (Wave 2 FG)   - Assessing available data (e.g. screening rates) and data reporting capacity (Wave 2 FG) - After onboarding by:   - Engaging staff at multiple levels (e.g. clinicians, chief medical officers, front-end staff) to complete readiness assessments (Wave 1 I)   - Helping recipients align CRCCP EBI implementation with existing initiatives in the clinic through RAs (Wave 1 I) |
| Implementation & Sustainment | | |
| Outer Context: Funding for Implementation Support | - Funding includes fiscal support provided by the system in which implementation occurs. Fiscal support can target multiple levels (e.g. staff training, fidelity monitoring/continuous quality improvement). | - CRCCP funding for implementation support facilitates sustained EBI implementation   - Ongoing TTA to clinic partners (Wave 1 and 2)   - Establishing or reinforcing QI practices to continually improve EBIs (Wave 1 FG, I) |
| Outer Context:  Inter-Organizational Environment and Networks | - Relationships of professional organizations through which knowledge of the EBI is shared and/or goals related to the EBI implementation are developed/established; includes inter-organizational collaboration | - Inter-organizational environment and networks inform type and amount of TTA to clinic partners   - Ongoing, informal touchpoints yield contextual information about clinic partners (Wave 3 FG)   - Periodic formal assessments evaluate EBI implementation quality, staff/ data capacity, clinic resources, workflows and screening processes (Wave 2 FG) |
| Bridging Factors:  Public Health-Primary Care Partnerships | - Bridging factors influence the implementation process as the inner context of organizations is influenced by the outer system in which the organization operates, and those influences are reciprocal. Bridging factors may include processes such as interagency collaboration, community-academic partnerships. | - Recipients prioritize health system and clinic partners with whom they have existing relationships (Wave 2 FG) - Recipients maintain relationships with clinics beyond CRCCP to monitor CRC screening prevalence and provide support for sustaining EBIs (Wave 1 FG, I) |
| Inner Context: Staffing Processes | - The processes or procedures in place at an organization related to the hiring, review, and retention of staff involved in the active delivery of the EBI and/or its implementation (e.g. professional training and qualification related to EBI delivery, staff turnover) | - Recipients collaborate with clinic partners on staffing processes to:   - Identify champion(s) who are passionate about CRC screening promotion and can support implementation of multiple screening programs (Wave 1 and 2 FGs)   - Establish EBI workflows and automated processes to mitigate challenges of clinic staff and champion turnover (Wave 2 FG)   - Identify co-champions or engage in succession planning while existing champions are still in place (Wave 2 FG) |

CRC = colorectal cancer; CRCCP = Colorectal Cancer Control Program; EBIs = evidence-based interventions; EPIS = Exploration, Preparation, Implementation, Sustainment (EPIS) Framework; FG = focus group; I = interview; QI = quality improvement; TTA = training and technical assistance.
