# Supplementary material for: Promising approaches to support sustained colorectal cancer screening promotion strategies in primary care clinics
Source: Prev Oncol Epidemiol. Author manuscript; Available in PMC 2025 Sep 9. (PMC12416315; doi:10.1080/28322134.2025.2512477)
Supplement: Schleuter_Supplement B [file NIHMS2099663-supplement-Schleuter_Supplement_B.docx]

**Supplementary Material B: Additional Illustrative Quotes by EPIS Construct and Finding**

| Finding | Illustrative Quote(s) |
| --- | --- |
| Inner Context: Leadership | |
| Recipients assess health system and clinic leadership support for implementing/enhancing CRC EBIs prior to partnering. | *There are multiple layers of leadership that has to be filtered down through an agreement—from the health system level to the clinic level, to the patient care level.* – **Wave 1 interview, Recipient 8, Health Department** |
| Inner Context: Health System and Clinic Organizational Characteristics | |
| Prior to onboarding health system and clinic partners, recipients assess health system and clinic capacity to implement/enhance EBIs and assess available data (screening rates, UDS measures) and health system/clinic capacity for reporting data. | *I see our role as going in and really helping clinics to solve a problem and improve a process. And a lot of times that seems to be around the EHR and their capacity to utilize it... my lack of knowledge of the clinical side and use of the EHR limits my ability to help them solve that problem technically...bringing in some EHR vendors like Athena Health, eClinicalWorks, Epic to really say, let's look at what it means do a workflow for documentation, for a stool- based test, Cologuard, and for colonoscopy referral, so that I can then say to people in the clinic who may be limited in their knowledge about how to utilize their EHR, this is how you do it.* – **Wave 2 FG, Recipient 9, University**  *We have to do a lot more outreach and relationship building within the health systems that have not participated in the CRCCP program before so that we can make sure that they're ready to participate when they want to...there's a lot of work within our rural health department getting connections with folks that aren't FQHCs and haven't learned how to do all this data reporting and may not have quite the capacity that the FQHCs have... And we're doing a lot of learning along the way as to what their challenges are specifically in the rural space compared with FQHCs. So relationship building, building a foundation, letting them know who you are, what you're doing what you can offer them, particularly when it comes to the leadership.* – **Wave 2 FG, Recipient 8, Health Department** |
| Once partners are onboarded: recipients engage health system/clinic staff at multiple levels to complete readiness assessments. Readiness assessments can help recipients align CRCCP EBI implementation with existing initiatives in the clinic and facilitate adoption of a sustainable workflow that aligns with how clinic care teams function. | *The medical providers are usually the ones that are more transient, especially in rural areas. So, we try to incorporate the whole medical team that are involved in this process, for example the CMO. That helps with buy-in. Usually the front-end staff are the lasting staff and the ones most invested in the community. They are the consistent piece of the puzzle. We focus on emphasizing that team-based approach.* – **Wave 1 interview, Recipient 1, Other**  *Some clinics prefer to talk to fill out some of the more in-depth sections of the readiness assessment tool. We found that to be useful and not as burdensome on them. Some people would rather have a conversation than type things out.* – **Wave 1 FG, Recipient 2, Other**  *Assessment tools are a starting point, but you have to continually check in. At the end of the day, when you are assessing readiness, you have to continue going back. There is incredible turnover. That is the nature of FQHCs. We always assess if there have been changes to capacity or readiness.* – **Wave 1 FG, Recipient 7, University** |
| Outer Context: Funding for Implementation Support | |
| CRCCP funding supports ongoing training and technical assistance to clinic partners to facilitate sustained EBI implementation. | *Sustainability requires continuous touch to maintain focus on EBIs*. – **Wave 2 interview, Recipient 7, University**  Example: It’s a challenge for these organizations to find funding elsewhere (i.e. beyond CRCCP) to sustain these EBIs/screening rates. – **Wave 3 interview, Recipient 8, Health Department**  Example: [FQHCs] are dependent on federal funding constantly. – **Wave 2 interview, Recipient 7, University** |
| CRCCP funding supports establishment / reinforcement of quality improvement practices within clinic partners. | *We did not start this way at the beginning, but we do spend a lot of time and effort educating now on quality improvement because generally the person we are most frequently in touch with at the clinic site is their QI person. But we also spend a lot of time during assessment trying to understand what they mean by that and what that person's experience and training is and what they've done around QI, etcetera. So, I think 100% building in that training and technical assistance about what is quality improvement, you know, and then this is the quality.* – **Wave 1 FG, Recipient 4, Health Department**  *We try new things. Sometimes they're brand new things, sometimes they're adaptations of existing things. We use data to help us determine whether to keep doing them, whether to stop doing them, or whether to tweak them and try it again.* – **Wave 1 interview, Recipient 2, Other**  *Once they got to the selection of EBIs, those one-hour sessions were spent planning their PDSAs [Plan-Do-Study-Act cycles] or discussing how things were going, additional data points that may need to be done, making those decisions of, did that work? Did that not?* – **Wave 1 interview, Recipient 7, University** |
| Outer Context: Inter-Organizational Environment and Networks, Including Formal and Informal Information Transmission | |
| Informal, consistent touchpoints with clinic partners give recipients contextual information to inform TTA. | *For an example, we learned that Exact Sciences, who does Cologuard, has a program that will provide free Cologuard to patients who are uninsured and are at 400% of the Federal poverty level or lower. And then we ended up discovering that there were [clinic] challenges with getting reports back from Cologuard because there was a lack of an interface between the Cologuard and Exact Sciences platform and the Athenahealth EHR. We learned about this through our informal meetings. We were able to set up some conversations with Exact Sciences and our clinic to figure out how to get this interface to work. So sometimes in those informal conversations...[things] pop up and then you realize, oh, that's a problem. How can we work on it together? So we appreciate those opportunities for sure.* – **Wave 2 FG, Recipient 9, University**  *After our TA with the clinic, we send the clinic teams a quick 3,4-question survey of how well the TA went, if they have additional questions, or would like additional support. So we actually gave the clinic and the staff the ability to report back to us in regard to how well they thought the TA went and if they needed additional support... We had a couple of times where we had a clinic saying, Yeah, we're still a little confused, or would like a little bit more hold-handing through this process. So we did schedule additional TA.* – **Wave 2 FG, Recipient 7, University**  *We have the benchmark of trying to achieve a screening rate at 15% above their baseline. But what we're trying to do right now is to measure what are the contextual factors that correlate or cause a higher screening rate return rate...how much does Administration participate in the program? Is there a champion? Are there medical assistants? ...And if those factors are in place do they tend to have a higher screening rate?* – **Wave 2 FG, Recipient 9. University** |
| Formal periodic assessments of EBI implementation quality, staff and data capacity, clinic resources, workflows, and screening processes inform the type and amount of TTA that clinic partners need. | *We created a very comprehensive project packet that lists all of the expectations... And then what our responsibilities are to them. So it's very clear it's in that packet. I will attach it periodically to our meetings just as a reminder. And then when we talk to them, we always let them know you have this coming up; you're going to have this survey coming up, or whatever. So we tried to be as clear as possible.* – **Wave 2 FG, Recipient 4, Health Department**  *We're always letting them know at multiple touchpoints what the expectations are... and we bring our evaluation partners in [university] and have them talk with [our health system and clinic partners] as well about what their expectations are. We make sure that it's very well spelled out in their scope of work in the contracting. And then we talk to them consistently about whether or not they're meeting or making the expectations.* – **Wave 2 FG, Recipient 8, Health Department** |
| Recipients conduct ongoing, focused meetings with clinic partners to provide TA, discuss specific objectives, and efficiently use clinic staff time. | *We have very specific monthly meetings, so when we're onboarding a clinic, we meet with them monthly, and each month there's a certain QI process that is going to take place during that meeting... It wasn't just necessarily a discussion toward EBI implementation. We used our [TA meetings] as work sessions. So that way, when we finished, and the clinic staff went back to their jobs. They didn't have this thing due to us the next month. We really used our [TA meetings] as working sessions, and we had specific details of which staff were required to attend each session.* – **Wave 2 FG, Recipient 7, University** |
| Bridging Factors: Partnerships | |
| Recipients prioritize onboarding health system and clinic partners with whom they have existing relationships and familiarity. | *I'm talking to our clinics pretty consistently throughout the projects... We have kind of a lot of things cooking with them all the time, so I can't really resonate as well with like this idea of prescreening in my mind because we're already working with them. And we understand them. ...when you already have pre-existing relationships with folks, I think that it actually makes the work a lot easier and very fulfilling as well.* – **Wave 2 FG, Recipient 1, Other** |
| Inner Context: Staffing Processes | |
| Recipients collaborate with health system and clinic partners to identify the right champion(s) to ensure CRC screening remains a focus among competing priorities. | *Sometimes [clinics] select someone who is maybe not the best provider champion. We've had folks select their CMO, but their CMO is a pediatrician and not particularly involved [in CRC]. I think we just need to give [clinics] more guidance. We would probably emphasize more strongly [that champions] at least need to see the age-eligible population*. – **Wave 2 FG, Recipient 4, Health Department**  *It speaks to the power of if you find the right person and get the spark... it's beyond just colorectal. It's that braiding that everyone talks about, you know, include the breast cancer screening with the colorectal cancer screening. And HPV, include all of the screening together, then all of them will increase. And so that is the role of champion...* – **Wave 2 FG, Recipient 3, University** |
| Recipients collaborate with clinic partners to establish EBI workflows and automate processes to mitigate challenges of clinic staff and champion turnover. | *[We’ve] really tried to build automated tools or really specific dashboards that people can use just kind of intuitively. If there are automated processes that reach out to patients, then, you know, that's not something that someone needs to be brought up to speed necessarily on. Those automations last throughout staff.* – **Wave 2 FG, Recipient 1, Other** |
| Health system and clinic partners can safeguard against stalled EBI implementation due to champion turnover by identifying co-champions or engaging in succession planning while existing champions are still in place. | *We have a clinic liaison who oversees all the clinics. So if one champion does go away, then that person could step in... I go along with having someone who could be the backup co-champion.* – **Wave 2 FG, Recipient 3, University** |

CRC = colorectal cancer; CRCCP = Colorectal Cancer Control Program; EBIs = evidence-based interventions; EHR = electronic health record; EPIS = Exploration, Preparation, Implementation, Sustainment (EPIS) Framework; FG = focus group; FQHC = federally qualified health center; HPV = human papillomavirus; QI = quality improvement; TA = technical assistance; TTA = training and technical assistance; UDS = uniform data system.
