# Supplementary material for: Promising approaches to support sustained colorectal cancer screening promotion strategies in primary care clinics
Source: Prev Oncol Epidemiol. Author manuscript; Available in PMC 2025 Sep 9. (PMC12416315; doi:10.1080/28322134.2025.2512477)
Supplement: Schleuter_Supplement C [file NIHMS2099663-supplement-Schleuter_Supplement_C.docx]

**Supplementary Material C: Top-Ranked Factors That Support Sustainment**


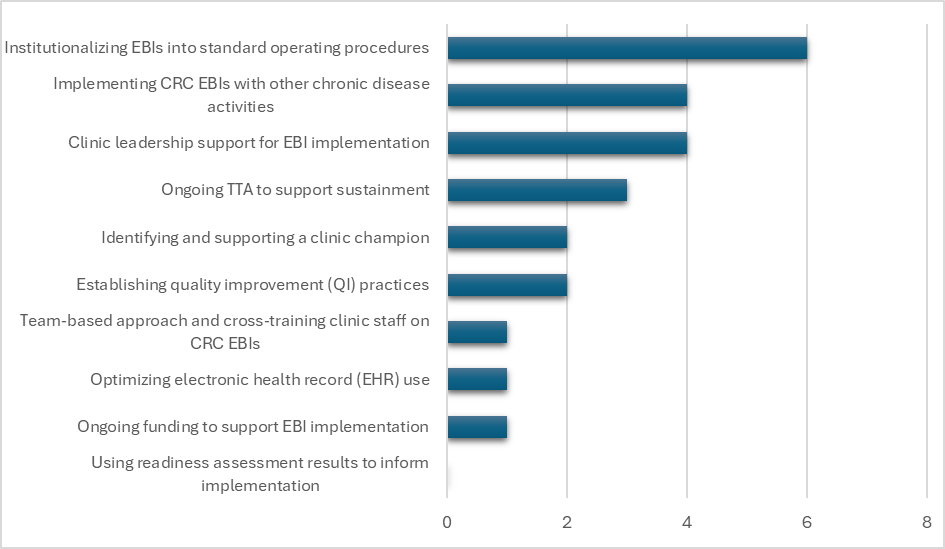


CRC = colorectal cancer; EBIs = evidence-based interventions; TTA = training and technical assistance
